# Neutrophil to lymphocyte ratio, a biomarker in non-muscle invasive bladder cancer: a single-institutional longitudinal study

**DOI:** 10.1590/S1677-5538.IBJU.2015.0243

**Published:** 2016

**Authors:** Vincenzo Favilla, Tommaso Castelli, Daniele Urzì, Giulio Reale, Salvatore Privitera, Antonio Salici, Giorgio Ivan Russo, Sebastiano Cimino, Giuseppe Morgia

**Affiliations:** 1 Sezione Urologia, Dipartimento di Chirurgia, Università di Catania, Italia

**Keywords:** Urinary Bladder Neoplasms, Neutrophils, Biomarkers

## Abstract

**Background:**

Bladder cancer represents one of the most important clinical challenges in urologic practice. In this context, inflammation has an important role in the development and progression of many malignancies. The objective of the present study was to evaluate the prognostic value of pre-treatment Neutrophil to lymphocyte ratio (NLR) on the risk of recurrence and progression in patients with primary non-muscle invasive bladder cancer.

**Materials and Methods:**

Data obtained from 178 bladder cancer patients who underwent transurethral resection of bladder tumor (TURB) between July 2008 and December 2014 were evaluated prospectively. NLR was obtained from each patient before TURB and defined as the absolute neutrophil count divided by the absolute lymphocyte count. Cox proportional hazards regression model was performed to calculate disease recurrence and progression including NLR.

**Results:**

During the follow-up study (median: 53 months), 14 (23.3%) and 44 (37.9%) (p=0.04) patients respectively with NLR<3 and ≥3experienced recurrence and 2 (3.3%) and 14 (11.9%) experienced progression (p=0.06), respectively. At the multivariate Cox regression analysis, NLR ≥3 was associated with worse disease recurrence (HR: 2.84; p<0.01). No association was found regarding disease progression. The 5-year recurrence free survival was 49% and 62% in patients with NLR≥3 and <3 (p<0.01). The 5-year progression free survival was 77% and 93% in patients with NLR≥3 and <3 (p=0.69).

**Conclusion:**

NLR predicts disease recurrence but not disease progression in NMIBC patients. NLR alterations may depend of tumor inflammatory microenvironment.

## INTRODUCTION

Bladder cancer represents one of the most important clinical challenges in urologic practice. At the time of initial diagnosis, approximately 70% of patients have cancers confined to the epithelium or the subepithelial connective tissue. In general, these cancers are primarily managed by endoscopic resection (TURB) (1–3). The dilemma in the management of non–muscle-invasive bladder cancer (NMIBC) still remain the risk of recurrence ranging from 30% up to nearly 80% and depending on the risk profile, up to 45% of tumors may progress to muscle-invasive disease within 5 years after initial diagnosis (4). To manage patients with NMIBC based on their individual risk, based on the course of the disease in well-controlled prospective randomized clinical trials, the European Organization for Research and Treatment of Cancer (EORTC) has developed risk tables to predict the individual risks for tumor recurrence or progression to muscle-invasive disease (4). According to the EORTC risk table, using a scoring system based on previous recurrence rate, tumor number, tumor diameter, T category, World Health Organization (WHO) grade, and the presence of concurrent carcinoma in situ (CIS), to estimate the risk of disease recurrence and progression at 1 and 5 years, patients with bladder cancer were stratified into low-, intermediate-, and high-risk group, which may guide clinical management (5). To further improve the predictive accuracy of risk Tables, a large number of clinical, molecular, biological, and environmental factors are available that have been studied in relation to bladder cancer development, recurrence, and/or progression in NMIBC.

In this context, inflammation has an important role in the development and progression of many malignancies (6). Putative mechanisms include the increased supply of factors that promote carcinogenesis and tumor progression by cells of the innate immune systems such as neutrophils and decreased anti-tumor response by immune cells of the adaptive system such as lymphocytes (7–11).

Moreover, the neutrophil to-lymphocyte ratio (NLR), which can easily be calculated from routine complete blood counts (CBCs) with differentials, is an emerging marker of host inflammation and it has been shown to be an independent prognostic factor for a variety of solid malignancies, including the urinary tract (12–14). Although a recent study found that preoperative NLR was associated with advanced pathologic stage at the time of cystectomy, as well as increased risk for disease recurrence, cancer-specific mortality and all-cause mortality (15), there are sparse and retrospective data on the prognostic role of NLR in patients with NMIBC. The purpose of our study was to evaluate the prognostic value of pre-treatment NLR on the risk of recurrence and progression in patients undergoing TURB for primary NMIBC.

## MATERIALS AND METHODS

Data obtained from 178 bladder cancer patients who underwent transurethral resection of bladder tumor (TURB) between July 2008 and December 2014 were evaluated prospectively after institutional internal review board approval was obtained. The diagnosis of bladder cancer was histologically confirmed by TURB in each patient. The clinical T stage of a bladder tumor was determined according to the 2002 Union International Contre le Cancer (UICC) TNM classification of bladder tumors. Tumor size was defined as the maximum tumor dimension estimated at the time of TURB and/or by clinical imaging. Tumors size were categorized in one group if its size was above 3cm and into another if below 3cm. The number and shape of the tumors were examined in the same manner. Concomitance of CIS was revealed in the surgical pathology of TURB. According to the pathology reports, patients were grouped as non-muscle invasive bladder cancer (NMIBC) or muscle-invasive bladder cancer (MIBC). Only patients with NMIBC were included in our study. Demographics and laboratory data, including hemoglobin (Hb) levels, platelet count, neutrophil count, lymphocyte count and serum values of neutrophil to lymphocyte ratio (NLR) were obtained from each patient before TURB. The NLR was defined as the absolute neutrophil count divided by the absolute lymphocyte count. Patient demographics, preoperative full blood count, operative details, and standard histologic tumor characteristics were recorded. Exclusion criteria for the present study were previous operation due to bladder tumor, ongoing treatment for bladder cancer, hematologic disorders or history of conditions that may have influenced blood cell lines such as connective tissue disease, presence of an active infection and/or immunodeficiency virus infection at the time of surgical intervention, prior or concomitant intravesical therapy with Bacille Calmette–Guérin (BCG), prior blood transfusion, and the presence of other cancer types or prior chemotherapy. Patients with non-urothelial cancer or for primary prostatic urothelial carcinoma were also excluded in order to maintain a homogenous cohort.

A second TURB was routinely performed in patients who had a T1 or high-grade tumor on initial TURB. Patients received post-operative intravesical instillations based on tumor characteristics, and at the discretion of the treating urologist. Postoperative follow-up consisted of cystoscopy and upper urinary tract imaging performed every three months for the first 2 years, every 6 months 2 to 5 years after surgery, and annually thereafter. Patients with a suspected recurrence underwent TURB. Disease recurrence was defined as the first pathologically confirmed tumor relapse in the bladder, regardless of the tumor stage. Disease progression was defined according to the International Bladder Cancer Group consensus definition for progression in NMIBC, in the presence of an increase in T category from CIS or Ta to T1 (lamina propria invasion), development of≥T2 or lymph node (N+) disease or distant metastasis (M1), or an increase in grade from low to high.

All participants provided written informed consent before enrolment and the study was conducted in accordance with regulatory standards of Good Clinical Practice and the Declaration of Helsinki (1996). The study was approved by our Institutional Research Ethics Committee.

### Statistical analysis

All statistical analyses were completed using SPSS versus 19 software (SPSS Inc, IBM Corp, Somers, NY, USA). The qualitative data were tested using the chi-square test or Fisher's exact test as appropriate and the continuous variables, presented as median, were tested by Mann-Whitney U-test.

The significance of the clinic and pathological variables associated with disease recurrence and progression were assessed using the Cox proportional hazards regression model, including age, stage, grade, tumor size, focality, BMI, gender, diabetes, Cis and NLR. Based on the ROC curve, we used a cut-off of 3 for the NLR with the best balance between sensitivity (50%) and specificity (76%) (area under the curve 0.60, 95% confidence interval [CI] 0.51-0.69; p<0.05).

Curves were tested with the log-rank test. Predictive accuracy of the model was assessed in term of the area under the curve (AUC) value, incorporating all significant and independent predictors. AUC values were also calculated by applying base model to the study cohort. The areas under the curve were compared via the Mantel-Haenszel test. For all statistical comparisons significance was considered as p<0.05. One thousand bootstrap resamples were used for all accuracy estimates and to reduce overfit bias. P-value<0.05 was considered as an indicator of statistical significance.

## RESULTS

Table-1 lists the baseline characteristics of the cohort. This study included 148 (83.1%) male and 30 (16.9%) female patients. The median age of all 178 patients enrolled in the study was 69.27 (IQR: 63.78-79.44), with a median follow-up of 53 months (IQR: 33.0-76.25). Patients with NLR≥3 were older (74.45 versus 67.94; p=0.02) and exhibited significant differences in term of pathological stage (26.6% versus 20.33%; p<0.05), number of multifocal tumors (53.4% versus 23.72; p=0.04) and Cis (50.0% versus 16.94%; p<0.05) if compared with those with NLR<3 (Table-2).

**Table 1:** Baseline characteristics of the patients.

		Total (n=178)
Age, year, median (IQR)		69.27 (63.78-79.44)
**Gender, no. (%)**		
	Female	30 (16.9)
	Male	148 (83.1)
BMI, median (IQR)		27.29 (24.2-29.4)
Hypertension, no. (%)		108 (60.7)
Diabetes, no. (%)		54 (30.3)
Dyslipidemia, no. (%)		36 (20.2)
NLR, median (IQR)		2.41 (1.69-3.62)
**Pathologic stage, no. (%)**		
	pTa	138 (77.5)
	pT1	40 (22.5)
**Pathologic grade, no. (%)**		
	Low grade	126 (70.3)
	High grade	40 (22.5)
**Concomitant CIS. no. (%)**		
	No	128 (71.9)
	Yes	50 (28.1)
**No. of tumours (%)**		
	1	118 (66.3)
	2-7	54 (30.3)
	≥ 8	6 (3.4)
**Tumour size, cm, no. (%)**		
	< 3cm	96 (53.9)
	≥ 3cm	82 (46.1)
**Smoking status**		
	Never	18 (10.1)
	Former	74 (41.6)
	Current	86 (48.3)
**EORTC Recurrence**		
	Low	48 (27.0)
	Intermediate	126 (70.3)
	High	4 (2.2)
**EORTC Progression**		
	Low	58 (32.6)
	Intermediate	82 (46.1)
	High	38 (21.3)

**IQR** = interquartile range; **BMI** = body mass index; **NLR** = neutrofil-to-lymphocyte ratio; **CIS** = carcinoma in situ

**Table 2:** Clinical characteristics of the patients according to neutrophil-to-lymphocyte ratio.

		NLR < 3 (n=118)	NLR ≥ 3 (n= 60)	p-value
Age, year, median (IQR)		67.94 (63.33-76.97)	74.45 (44.75-80.41)	0.02
**Gender, no. (%)**				
	Female	20 (16.9)	10 (16.7)	
	Male	98 (83.1)	50 (83.3)	
BMI, median (IQR)		27.29 (24.22-32.44)	27.15 (24.20-28.39)	0.44
Hypertension, no. (%)		70 (59.3)	38 (63.3)	0.60
Diabetes, no. (%)		32 (27.1)	22 (36.7)	0.19
Dyslipidemia, no. (%)		22 (18.6)	14 (23.3)	0.46
NLR, median (IQR)		1.94 (1.57-2.41)	4.02 (3.55-4.82)	<0.01
Pathologic stage, no. (%)				0.03
	pTa	103 (87.28)	44 (73.3)	
	pT1	15 (12.72)	16 (26.6)	
**Pathologic grade, no. (%)**				0.22
	Low grade	90 (76.27)	36 (60.0)	
	High grade	28 (23.73)	24 (40.0)	
**Concomitant CIS, no. (%)**				0.01
	No	98 (83.05)	24 (40.0)	
	Yes	20 (16.94)	36 (60.0)	
**No. of tumours (%)**				0.04
	1	90 (76.28)	28 (46.6)	
	2-7	28 (23.72)	26 (43.4)	
	≥ 8	0 (0)	6 (10.0)	
**Tumour size, cm, no. (%)**				0.60
	< 3 cm	67 (56.78)	29 (48.33)	
	≥ 3 cm	51 (43.22)	31 (51.66)	
**Smoking status**				0.06
	Never	12 (10.2)	6 (10.0)	
	Former	63 (53.4)	11 (18.3)	
	Current	43 (36.4)	43 (71.7)	
**EORTC Recurrence**				0.34
	Low	32 (27.1)	16 (26.7)	
	Intermediate	86 (72.9)	40 (66.6)	
	High	0 (0.0)	4 (6.7)	
**EORTC Progression**				0.15
	Low	39 (33.0)	19 (31.7)	
	Intermediate	62 (52.5)	20 (33.3)	
	High	17 (14.5)	21 (35.0)	
**Chemotherapy instillation**		90 (76.27)	40 (66.6)	0.17
**Immunotherapy instillation**		10 (8.5)	9 (15.0)	0.18

**IQR** = interquartile range; **BMI** = body mass index; **NLR** = neutrofil-to-lymphocyte ratio; **CIS** = carcinoma in situ

**Figure 1:**
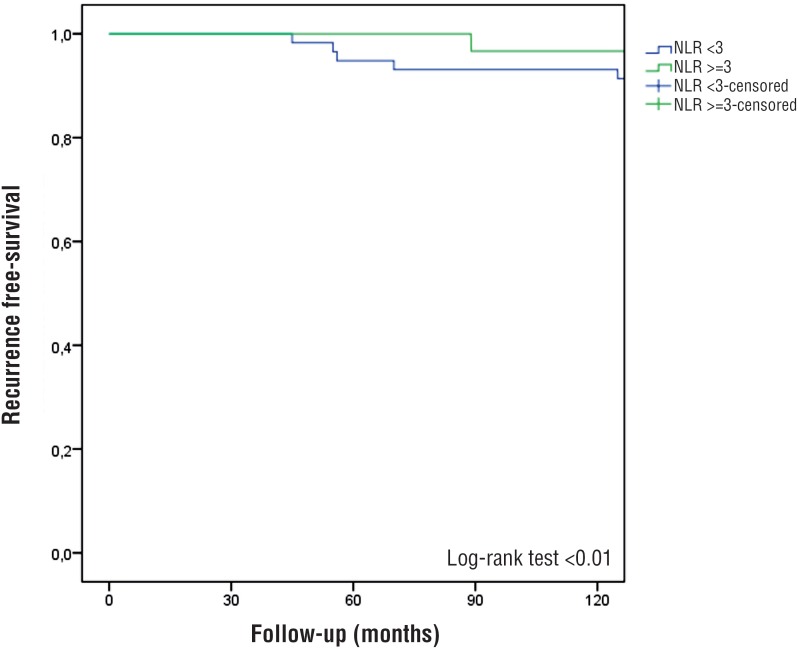
Kaplan-Meier estimates of recurrence-free survival stratified by NLR.

During the follow-up study, 14 (23.3%) and 44 (37.9%) (p=0.04) patients respectively with NLR<3 and ≥3 experienced recurrence and 2 (3.3%) and 14 (11.9%) experienced progression (p=0.06), respectively. At the multivariate Cox regression analysis, NLR≥3 was associated with worse disease recurrence (HR: 2.84 [IQR: 1.505.75]; p<0.01). Pathological stage pT1 (p<0.01), high grade (p<0.01), no. of tumors (p<0.01) and smoking status (p<0.01) were independently predictors of disease recurrence. No association was found between NLR≥3 and disease progression at the multivariate Cox regression analysis (Table-3). The 5-year recurrence free survival was 49% and 62% in patients with NLR≥3 and <3 (p<0.01) (Figure-1). The 5-year progression free survival was 77% and 93% in patients with NLR≥3 and <3 (p=0.69).

**Table 3:** Multivariate Cox-regression analysis for predictors of disease recurrence and progression adjusted for BMI and intravesical therapy.

		Disease Recurrence		Disease Progression	
		HR (95% CI)	p-value	HR (95% CI)	p-value
Age, year				1.02 (0.94-1.11)	0.60
Gender, female vs. male		0.48 (0.15-1.53)	0.22	0.30 (0.10-0.50)	0.98
Diabetes, yes vs. no		1.25 (0.64-2.42)	0.50	1.20 (0.19-7.54)	0.84
**Pathologic stage**					
	pTa	1.00 (Ref.)		1.00 (Ref.)	
	pT1	4.18 (1.87-9.35)	<0.01	5.75 (0.54-60.99)	<0.05
**Pathologic grade**					
	Low grade	1.00 (Ref.)		1.00 (Ref.)	
	High grade	2.88 (1.33-6.18)	<0.01	2.96(0.46-18.86	<0.05
**Concomitant CIS**					
	No	1.00 (Ref.)		1.00 (Ref.)	
	Yes	2.20 (0.82-5.88)	0.11	7.27 (1.01-14-76)	<0.05
**No. of tumours**					
	1	1.00 (Ref.)		1.00 (Ref.)	
	2-7	2.25 (1.25-4.07)	<0.01	5.33 (0.69-41.25)	0.11
	≥ 8	1.62 (0.67-7.12)	<0.01	0.98 (0.60-1.20)	0.98
**Tumour size, cm**					
	< 3 cm	1.00 (Ref.)		1.00 (Ref.)	
	≥ 3 cm	1.57 (0.84-2.92)	0.15	2.26 (0.42-12.27)	0.34
**Smoking status**					
	Never	1.00 (Ref.)		1.00 (Ref.)	
	Former	3.13 (1.20-8.78)	<0.01	1.14 (0.30-3.04)	<0.05
	Current	1.78 (0.95-3.30)	<0.01	5.62 (1.32-9.45)	<0.05
**NLR**					
	< 3	1.00 (Ref)		1.00 (Ref.)	
	≥ 3	2.84 (1.50-5.75)	<0.01	5.35 (0.39-73.70)	0.21

The bootstrapping calculations generally confirmed the p-values of the conventional Cox-regression analysis with larger ranges of 95% CI of the ORs (data not shown). After one thousand bootstrapping resampling, the derived ROC of the base model was 0.75, while when incorporating the NRL to this model the derived AUC value resulted in 0.78. However, the gain in accuracy (3%) was not statistical significant.

## DISCUSSION

Non-muscle invasive bladder cancer (NMIBC) represents a heterogeneous group of tumors with different rates of recurrence, progression, and disease-related mortality. NMIBC are initially treated with TURB after which adjuvant therapy should be considered according to tumor-based risk stratification that helps identify the appropriate treatment for each group of patients based on their risk for recurrence or progression (3). To further improve the ability to select the appropriate treatment for each individual patient, especially in doubtful cases such as patients at intermediate or high risk for recurrence or progression, additional, independent pretreatment predictors of outcome may help to further individualize treatment options within each risk group (5, 16). In recent years, the host inflammatory response has gained increasing attention in oncology research. In fact, increasing evidence showed the association of inflammation and cancer. While initially thought to represent an anti-tumor response, immune cells, particularly those of the innate immune system, also exhibit effects that promote carcinogenesis and cancer progression. Proposed mechanisms include increased supply of growth factors, survival factors, pro-angiogenic factors, extracellular matrix-modifying enzymes and inductive signals that may lead to epithelial-to-mesenchymal transition (7, 17). Recently, several inflammatory parameters obtained from blood tests, including C-reactive protein, NLR, platelet-lymphocyte ratio, and albumin levels, were associated with the treatment outcome of several malignancies (18). In this context, there is a biological rationale for using NLR, the ratio of circulating neutrophils (immune cells of the innate system) to lymphocytes (immune cells of the adaptive system), as a measure of the systemic host response when evaluating the association between inflammation and cancer outcomes. In fact, an enhanced neutrophil response and/or suppression of lymphocyte leading to a high NLR might promote carcinogenesis and inhibit anti-tumor immune response (18, 19). Several studies have shown that a pretreatment high NLR was associated with worse disease-specific and overall survival in muscle-invasive bladder cancer and the upper urinary tract (14, 15, 18, 20). Similarly, a correlation between high NLR levels and muscle-invasive disease at TURB was found by other studies (2, 18). The role of negative predictor of recurrence-free survival and cancer specific survival of elevated NLR was also confirmed in a recent meta-analysis including 17 studies involving 3159 cases with urinary cancers (21). However, most previous studies which evaluated the predictive value of NLR in bladder cancer, were retrospective and included a mixed and heterogeneous group of patients with muscle-invasive and high risk non-muscle invasive tumors. Besides heterogeneity, divergence may result from many other factors, including age distribution, gender, lifestyle and so on. Herein we found an association between high NLR levels and worse disease recurrence-free survival even after adjustment for common risk factors but not for progression free-survival as recently reported by a retrospective study (18). These findings are consistent with previous reports that found an association between greater NLR and unfavorable tumor characteristics, worse recurrence-free, disease specific and overall survival in patients with muscle-invasive bladder cancer or high-risk patients with NMIBC (2, 18). However, on the contrary to recent report (18), our study did not found association between higher NLR and disease progression at the multivariate analysis. We recognize that our study is limited by its small sample size and non-randomized nature. Furthermore, we acknowledge the relative arbitrary cut point of NLR ratio used for the analyses in our study, nevertheless, this threshold allows our data to be contextualized in light of previously published analyses, which, likewise, dichotomized NLR (2, 7, 15). In addition, despite the use of standard treatment protocols, information regarding the use of intra-vesical maintenance treatment, which may have influenced outcomes, was lacking. Furthermore, Ta and T1 category tumors are distinct diseases that may be associated in a different manner with NLR. Larger cohorts are required to evaluate NLR separately in both these groups. Finally, we recognize that these data are from a single, tertiary referral institution and, as such, require external validation. Nevertheless, within the limitations of a nearly-phase study for marker assessment, our findings suggest that NLR is a potential prognostic marker for prediction of disease recurrence in NMIBC and may better risk-stratify patients in the pre and postoperative settings in order to guide treatment strategies.

It could be also postulated that NLR alterations in NIMBC patients mainly depends of tumor inflammatory microenvironment and cancer biology raising new opportunities for therapeutic interventions (22). The near future of NLR application could be also directed into the interpretation of immune response of BCG therapy. It should be in fact taken into account that NLR is an expression of the immune system, a marker that can be also associated with immunotherapy and considered as a potential predictive factor of BCG response.

Further prospective, well-controlled clinical studies of diverse patients in multiple institutions are required to validate the role of NLR as a prognostic marker, which may improve current risk stratification tools and treatment outcome in this group of patients.

## CONCLUSIONS

In conclusion, NLR is an inexpensive hematologic test based on commonly measured parameters that may predict disease recurrence, T1 category, concomitant Cis and high tumor grade in a cohort of patients with NMIBC. Whereas our results suggest that NLR may have a role as a prognostic biomarker, further studies are needed to maximize the clinical utility of NLR in patients with NMIBC.

## ABBREVIATIONS

NLR: = Neutrophil to lymphocyte ratio
NMIBC: = Non muscle invasive bladder cancer
TURB: = Transurethral resection of bladder tumor
AUC: = Area under the curve
